# Editorial: Antibiotics in engineered and natural environments: occurrence, fate, kinetic and microbial impact

**DOI:** 10.3389/frabi.2024.1437802

**Published:** 2024-06-07

**Authors:** Ilke Pala-Ozkok, Tugce Katipoglu-Yazan

**Affiliations:** ^1^ Department of Chemistry, Bioscience and Environmental Engineering, Faculty of Science and Technology, University of Stavanger, Stavanger, Norway; ^2^ Environmental Engineering Department, Faculty of Civil Engineering, Istanbul Technical University, Istanbul, Türkiye

**Keywords:** antibiotic resistance, wastewater treatment, antibiotic pollution, multidrug resistance, cost benefit analysis, activated sludge

Antibiotics are the most powerful tools available for the treatment of infectious diseases. In addition to being used for the treatment of infections in human patients and farm animals, antibiotics are also routinely given to healthy farm animals to promote growth and proactively prevent disease outbreaks.

The administered antibiotics and their metabolic by-products pass through human and animal bodies and ultimately enter the environment. Moreover, emissions from production facilities are additional causes of pollution. Since the wastewater treatment plants are not designed to remove antibiotics and their by-products from the wastewater, trace amounts of antibiotics reach receiving water bodies.

Although antibiotics exist in trace concentrations in water and wastewater, they pose a potential hazard, including the development of antibiotic resistance due to long-term exposure to sub-inhibitory concentrations, contamination in the environment, and threat to ecological safety and human health. Therefore, it is urgent to mitigate the problem of antibiotic contamination in water environments.

This Research Topic focused on the occurrence and transformation of antibiotics in the environment and also treatment of antibiotic-contaminated waters and wastewaters together with kinetic analysis of treatment methods. Another focus point was also microbial impact of antibiotics on these treatment systems and natural water bodies, including antimicrobial resistance.

Azabo et al., aimed to assess the economic implications of reducing antimicrobial use on Tanzanian broiler farms, where antimicrobial use for infection prevention is required due to rapidly increasing demand for poultry meat leading to intensified production methods. Results showed that reducing antimicrobial use led to increased production costs, which was also confirmed by modeling studies, but also reduced disease costs significantly. However, achieving this reduction relies on the availability of effective alternatives for disease control. The authors recommended future study on impact of antimicrobial use reduction on morbidity and mortality and the efficiency of additional control and other measures of producing poultry meat without high concentrations of antibiotics.

Walana et al., studied the antimicrobial resistance pattern in Ghana’s Bono East Region. This study employed the WHO’s AWaRe (ACCESS, WATCH, and RESERVE) antibiotic classification and the European Centre for Disease Prevention and Control (ECDC)’s multi drug resistance (MDR) definition for antimicrobial resistance (AMR) isolates from clinical specimens. Methodologically, bacterial culture and antibiotic sensitivity test results were reviewed and categorized according to the AWaRe and ECDC-MDR classifications. Analysis of 3,178 clinical specimens revealed a notable presence of pathogens, particularly in samples from female patients, with 37.4% yielding isolates. Findings indicated varying levels of susceptibility and resistance among different antibiotic classes, with Gram-positive isolates demonstrating higher resistance to certain antibiotics compared to Gram-negative isolates. Notably, a proportion of isolates exhibited MDR characteristics, albeit relatively low according to the ECDC definition. The study underscores the need for further research to establish national criteria for MDR in Ghana and to address the diverse and concerning spectrum of AMR.

Pala-Ozkok et al., studied the acute and chronic exposure of sulfamethoxazole on the kinetics and microbial structure of an activated sludge system. The authors based their study on respirometric analysis and model evaluation of the oxygen utilization rate profiles. The results showed that, chronic exposure to sulfamethoxazole resulted in inhibition of substrate storage, doubling of endogenous decay rates, together with mild inhibition on the growth and hydrolysis kinetics. Additionally, activated sludge modeling results showed that sulfamethoxazole had a binding impact on the available organic carbon resulting in less oxygen consumption throughout the process. Using DNA sequencing and antibiotic resistance gene analyzes, the study also showed that chronic exposure to sulfamethoxazole caused a change in the community structure at species level. The bacterial community after the chronic exposure was shown to be dominated by resistant species like *Arthrobacter* sp and members of *Chitinophagaceae* and *Intrasporangiaceae* families. In addition to acute and chronic impact the study also investigated the intermittent exposure impact, which indicated a drop in the severity of the impact after the 20 days of intermittence.

Rathinavelu et al., mapped and reviewed the data on antibiotics from natural and engineered water environments in India which is amongst largest antibiotic consumers and producers. The distribution and occurrence of antibiotics in Indian water environments such as wastewater, sewage sludge, treatment plants, surface waters including rivers, lakes, and reservoirs, groundwater and drinking water were investigated. Several factors including population density, sewage or other point sources, variations in sewage treatment practices, precipitation and climate were included in the study. Systematic analyses of reported data were performed between 2000 and 2023 years with a selected set of keywords such as antibiotic, antimicrobial resistance, water, wastewater, sewage, sewage water, surface water, drinking water, and India. The authors highlight emergence of antibiotic pollution and subsequent risk of AMR development following evaluation of scarce data. They recommend a framework for effectively monitoring antibiotic pollution in India and directions for future research. The importance of increasing public awareness on the antibiotic consumption was also emphasized.

Basiry et al., studied several wastewater treatment plants (WWTPs) in Norway with different wastewater characteristics, process configurations, and generated from different catchment area activities. Selected antibiotic resistance genes (ARGs) from literature such as blaSHV-1, blaTEM-1, msrA, ermA, ermC, tetM, tetL, tetA, vanA, and vanC were monitored in four wastewater treatment plants for two seasons by PCR analyses. The obtained monitoring results from the influent, effluent and sewage sludge revealed that ermC, tetA, and tetM were present in all lines of wastewater treatment plants. The results also pointed out that commonly observed blaSHV-1, blaTEM-1, tetA, vanA, and vanC were not detected in any of the samples. Regardless of the origin of wastewater minimum inhibitory concentration (MIC90) for ampicillin, vancomycin, and tetracycline were determined as >128, ≥128, and 32 µg/mL, respectively. Bacterial community compositions identified in different treatment plants shared similarities with other wastewater treatment plants located in different regions of the world. The authors concluded that WWTPs do not significantly reduce ARGs in wastewaters and new ARG removal technologies need to be developed. They also hypothesize that environmental factors such as temperature may influence ARGs more than antibiotic concentrations or wastewater origin. In this process vertical gene transfer rather than horizontal gene transfer may be responsible which need to be further investigated.

In conclusion, this Research Topic provides an overview of occurrence and transformation of antibiotics in the engineered and natural aquatic environment. Different studies have been collected in this Research Topic which analyzed different systems and worked with different antibiotic concentrations characterizing both industrial and domestic effluents, focusing on different regions in the World. Therefore, this Research Topic presents a valuable reference for future studies on antibiotic resistance in the environment.

## Author contributions

IP-O: Writing – original draft, Writing – review & editing. TK-Y: Writing – original draft, Writing – review & editing.

